# On the Value of Albumen in the Urine, &c.

**Published:** 1841-10

**Authors:** W. C. Roberts

**Affiliations:** New York


*On the value of Albumen in the Urine, as indicative of Renal Disease.*—By W. C. Roberts, M. D., of New York. It has been, and still is by many supposed, that the presence of albumen in the urine is always a sure sign of the existence of renal disease. Upon this subject much misconception exists, and it is important to set the matter in its proper light.

It is incorrect to hold, as some do, that albuminous urine is pathognomonic. or, by itself characteristic, of granular degeneration of the kidney, or Bright's disease. A variety of other causes, as a blister, certain indigestible articles of food, as pastry, cheese, &c., hæmaturia, the existence of pregnancy, the action of mercury, and a variety of local irritating causes, will render the urine albuminous.

In reference to this matter, Dr. Bright, the distinguished discoverer of the disease which bears his name, observes: "I am anxious to explain my view, on an important point, connected with the disease attending albuminous urine, in reference to which I have been singularly misunderstood, and as a necessary consequence, misrepresented, by many who have written and lectured on this interesting subject. The misconception to which I refer is, that I maintain the occurrence of albuminous urine to be always, and necessarily, connected with that organic disease, which, in its various shapes and modifications, has been so fully described. Now the truth is, that I have never written upon the subject, *without studiously stating the contrary*, and declaring, that I considered the disease, in its commencement, *entirely functional.* In my second volume, I thought it right to say: 'from the observations of some of my professional brethren, I am led to suppose they consider me as asserting, that this description of urine exists *only* when organic disease has already taken place in the kidney. This, however, is by no means the view which I have taken of the subject. I believe functional disease in this, as in most other cases, precedes the structural change for even many weeks and months, and that the kidnies of a patient who has been cut off early by some other disease, may afford very little evidence of diseased structure.'"

Dr. Graves, of Dublin, and Dr. Elliotson, of London, have been the most distinguished among the opponents of the idea improperly, as it thus appears, ascribed to Dr. Bright, of the universality of the connexion between albuminous urine and diseased kidney. The former states distinctly that he cannot admit that the albuminous state of the urine in dropsy, depends upon an alteration in the texture of the kidney; having so often seen cases in which the albumen disappeared under suitable treatment, that it must depend upon functional disorder. The reader will find some remarks by him on this subject, in the Dublin Journal; and in the American Journal of the Medical Sciences, for Feb., 1839, he will find a clinical lecture of his, in which he details the case of a man who had dropsy with albuminous urine, who recovered—his urine becoming healthy, and so continuing for a fortnight. At the end of this time he got erysipelas of the head and face, and his urine became albuminous again. He died, and on examination, his kidneys were found large, pale, rather soft, but not granular. From this case he draws the conclusion, that the general state of the constitution influences the appearance of albumen in the urine, more than any change in the structure of the kidney. He cites another case by Morison, where a man passed albuminous urine for five years, and whose kidneys, except that they were blanched, were natural. He then mentions a case, where a boy, after scarlatina, passed highly albuminous urine, whose kidneys were found healthy after death: and he says that Forget has recorded many others. He further adds, that the albuminous state of the urine appears to him to be the *cause* of Bright's disease. And he conceives that a deposition of albuminous molecules, separated by coagulation, remain in the secreting tubes of the kidneys, which they gradually fill and distend, and thus give rise to an obliteration of the tissue, which is called '*Morbus Brightii.*' The kidneys are thus not the cause of the secretion, but themselves the receptacles of it.

This ingenious theory is founded on a microscopical examination, made by Valentine. It is, however, only a single case of one form of the disease, and is controverted by subsequent examinations of Gluge's.

Dr. Elliotson remarks, that because the urine is albuminous, it cannot, he thinks, be inferred, that the kidney is in a state of organic disease; "for," says he, "I have seen so many dropsical persons, who had albuminous urine, restored to perfect health, that I could not suppose the kidney to have been organically diseased. Nor can I admit," he continues, "that there existed in these cases, inflammation or congestion of the kidneys, because I did not observe the signs of those affections." Lastly, he has, he says, seen the urine albuminous when there existed no reason to suspect a disease of the kidney, and although, in a state of disease or congestion of the kidney, the urine may be generally albuminous, he should not, because it was so, conclude the kidney diseased.

Not to cite any of those cases particularly, in which it is said that albuminous urine has been met with accompanied by renal disease, it may be stated, in a general way, that some are of dubious authenticity, or import, being scarcely free from the suspicion that they were, in some measure, connected with the morbid action which gives rise to granular deposition; and the whole taken together, will merely show, that beside granular degeneration, *some other diseases may, in a few, comparatively very rare instances, be accompanied with the discharge of albumen in the urine,* the affection being then, as admitted by Bright, functional only. The knowledge of this fact is certainly useful in guarding us against that unreservedly discouraging opinion, which without it we might feel always disposed to give, both as to the probable supervention of dropsy, when it had not yet occurred, and the almost certainly fatal result when it had. But, unquestionably, there is no other cause, or rather, there are no other causes taken together, by which an albuminous impregnation is so often induced, as by the disorder in question; and it may, in the present state of our knowledge, be stated as a general proposition, that the excessive loading of the urine with albumen is characteristic of granular degeneration of the kidney. In no case of this disease hitherto noticed, has the urine failed, at some time or other, to be albuminous.

Besides the diseases which have been already noticed, albumen is found in the urine as a consequence of certain organic diseases of the kidney also; these are pyelitis, chronic and acute nephritis, encysted states of the kidney, cancer, tubercle, cerebriform cancer, strumous degeneration, and diabetes.

The three following propositions embody the sum of our knowledge, with respect to the albuminous urine as a symptom of renal disease. 1st. Whenever its exists in the urine, there exists in all probability, a lesion of the genito-urinary apparatus. 2d. That the existence of such urine, of a diminished specific gravity, in a person presenting, as yet, no other symptoms of disease, is sufficient to indicate the existence of renal affection, and to prognosticate the supervention of dropsy if the affection proceeds. The exceptions to this general rule are, that the renal affection may be functional only, or be some other of the morbid states of the kidney, in process of development. 3rdly. That if a pale and turbid urine be passed, for the most part, depositing no sediment, giving by appropriate test an abundant albuminous coagulum, containing or not other elements of the blood, having a specific gravity below the normal standard, with a co-existing diminution of the urea, uric acid, urates and phosphates, and there be *general anasarca*, with or without fever, or pain in the loins, there exist some of the anatomical characters which constitute albuminous nephritis. (*Rayer*, *Christison*.)—*N. Y. Med. Gazette.*

